# Evidence of cryptic and pseudocryptic speciation in the *Paracalanus parvus* species complex (Crustacea, Copepoda, Calanoida)

**DOI:** 10.1186/1742-9994-11-19

**Published:** 2014-03-02

**Authors:** Astrid Cornils, Christoph Held

**Affiliations:** 1 Alfred-Wegener-Institut, Helmholtz-Zentrum für Polar- und Meeresforschung, Am Alten Hafen 26, D-27568 Bremerhaven, Germany

**Keywords:** Copepoda, *Paracalanus parvus*, Cryptic species, Pseudocryptic species, Phylogeography

## Abstract

**Introduction:**

Many marine planktonic crustaceans such as copepods have been considered as widespread organisms. However, the growing evidence for cryptic and pseudo-cryptic speciation has emphasized the need of re-evaluating the status of copepod species complexes in molecular and morphological studies to get a clearer picture about pelagic marine species as evolutionary units and their distributions. This study analyses the molecular diversity of the ecologically important *Paracalanus parvus* species complex. Its seven currently recognized species are abundant and also often dominant in marine coastal regions worldwide from temperate to tropical oceans.

**Results:**

COI and Cytochrome *b* sequences of 160 specimens of the *Paracalanus parvus* complex from all oceans were obtained. Furthermore, 42 COI sequences from GenBank were added for the genetic analyses. Thirteen distinct molecular operational taxonomic units (MOTU) and two single sequences were revealed with cladistic analyses (Maximum Likelihood, Bayesian Inference), of which seven were identical with results from species delimitation methods (barcode gaps, ABDG, GMYC, Rosenberg’s P(AB)). In total, 10 to 12 putative species were detected and could be placed in three categories: (1) temperate geographically isolated, (2) warm-temperate to tropical wider spread and (3) circumglobal warm-water species.

**Conclusions:**

The present study provides evidence of cryptic or pseudocryptic speciation in the Paracalanus parvus complex. One major insight is that the species *Paracalanus parvus* s.s. is not panmictic, but may be restricted in its distribution to the northeastern Atlantic.

## Introduction

### Species delimitation and DNA barcoding

Species delimitation is a necessary process to study the life history and ecology of marine planktonic organisms, but its preciseness is dependent on the prior taxonomic knowledge. Detailed taxonomic keys may not be available for a specific region making it difficult to evaluate whether the studied specimen belongs to an already described species or an unknown species. In copepods identification is often based on only a few diagnostic characters due to the high abundances and the necessity to classify thousands of organisms [1]. These characteristics are mostly developed only in adult organisms often making it nearly impossible to identify juveniles, which are regularly more abundant than adults. Furthermore, morphological differences between sibling species may only be inconspicuous or non-existent (pseudocryptic and cryptic speciation) and thus, species may be overlooked. This phenomenon has been observed in many marine organisms (e.g. [2-4]). Cryptic speciation may be more prevalent in the marine realm than in terrestrial habitats [5].

These observations imply that traditional species concepts based on morphologically identified marine taxa may have greatly underestimated species richness [6]. Also, genetically divergent yet morphologically similar species may differ in their ecological and behavioural adaptations [7]. In general, barriers to gene flow in marine pelagic systems can often not be clearly identified (e.g. [8,9]). To overcome these obstacles, DNA barcoding with mitochondrial gene fragments has been successfully used for species discrimination in marine plankton (e.g. [10-12]). Initially, DNA analysis using “barcoding gaps” was based on genetic distances between *a priori* defined groups and did not take into account differences in divergence times between species or other taxa and thus is questioned to be useful for DNA taxonomy (e.g. [13,14]). However, a number of methods to measure species delimitation including DNA barcoding without defining prior groups have been published (e.g. [15-18]) These methods will be applied in the present study.

### *Paracalanus parvus* species complex

Species of the *Paracalanus parvus* complex are abundant in many marine ecosystems from temperate to tropical regions (e.g. [19-21]). Extensive research has provided valuable information on the feeding and reproduction biology of *Paracalanus parvus* (e.g. [22-24]). However, the taxonomy and species distribution of this species complex is not well understood. Currently the *P. parvus* complex consists of seven species: *P. parvus*, *P. indicus*, *P. quasimodo*, *P. nanus*, *P. intermedius*, *P.tropicus*, and *P. serrulus*. The latter may possibly belong to the *Paracalanus aculeatus* species complex [25], and *P. intermedius* may be a junior synonym of *P. parvus*[26]. The circumglobal distribution of *P. parvus* has also been questioned e.g. [24].

In the present study mitochondrial marker genes (cytochrome *c* oxidase subunit I (COI), cytochrome *b* (Cytb)) will be applied to investigate the genetic diversity of this species complex. Genetic markers are favourable as a tool to distinguish between species compared to microscopy considering that exact morphological identifications can be nearly impossible if morphological characteristics, such as antennules or exopods of swimming legs, are missing due to net sampling. We aim to define molecular operational taxonomic units (MOTUs; [27]) within the *P. parvus* species complex to establish a framework for future studies, and to elucidate their geographic boundaries.

## Material and methods

### Preservation and morphological identification

**Figure 1:**
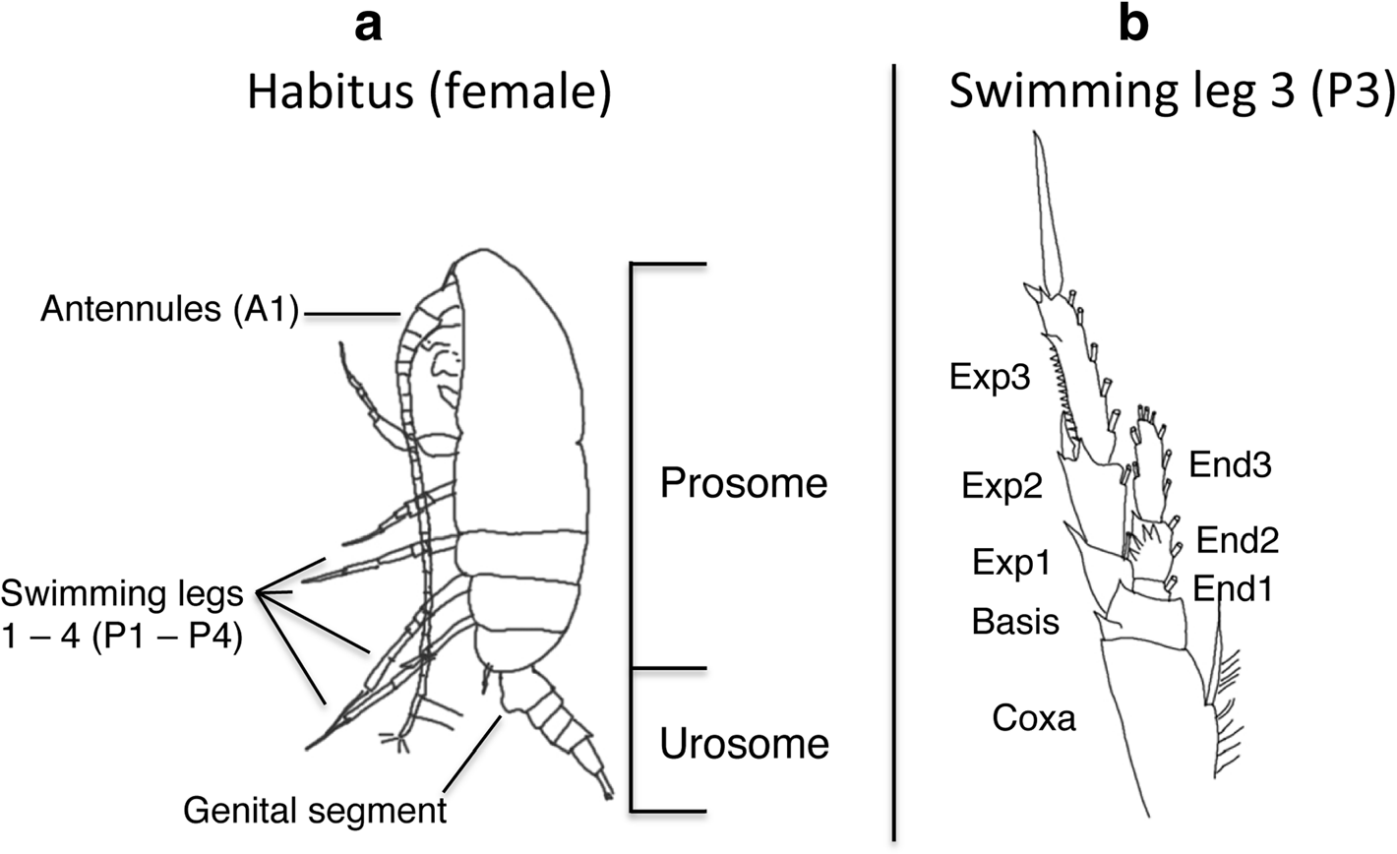
**Female specimen from the Gulf of Maine (NWA) with the important body parts for identification. a**. habitus, **b**. swimming leg 3 (Exp: Exopod, End: Endopod).

A total of 162 females of the *Paracalanus parvus* species complex from 44 samples were analysed (Additional file 1). Specimens were preserved in 96% pure ethanol with a change in ethanol after 24 hours of the initial fixation. Individuals of the *P. parvus* species complex were separated from other *Paracalanus* species such as *P. aculeatus* or *P. denudatus* due to the differences in segmentation and length of the antennules, the form of the spermatheca and the length of the inner setae on the caudal rami. Furthermore, the total length (TL), the prosome:urosome ratio (P:U), the length of the antennules (A1) relative to TL, and the shape of the forehead were noted prior to the DNA extraction (Figure 1, Additional file 2). However, some important diagnostic morphological characters can only be seen using light microscopy. Therefore, specimens from each sampling location were set aside as paratypes for detailed morphological analysis (according to e.g. [28-30]) and as para-vouchers preserved in ethanol. The latter are stored in the cooling facilities of the Alfred-Wegener-Institut. Specimens from Chinese coastal waters (Yellow Sea) were available from samples preserved in formalin and used for morphological identification only, while sequences from this region were obtained from GenBank (Table 1).

**Table 1:** **Information on 43 COI sequences of the *Paracalanus parvus *** **species group published in GenBank**

**Submitted species name**	**Accession numbers**	**Location**	**Submitted by**	**Haplotypes**
*P. parvus*	KC287780	Gulf of Maine	Blanco-Bercial et al. (2013) unpubl.	NWA4
*P. parvus*	EU599546	Chinese coastal waters	Sun S, Wang M and Liu B (2008) unpubl.	NWP1
*P. parvus*	KC287784	Japan Sea, Iki Island	Blanco-Bercial et al. (2013) unpubl.	NWP1
*P. parvus*	AF474110	Japan Sea, Iki Island	Bucklin A and Frost BW (2002) unpubl.	NWP2
*P. parvus*	KC287781	Japan Sea, Iki Island	Blanco-Bercial et al. (2013) unpubl.	NWP2
*P. parvus*	KC287787	Japan Sea, Iki Island	Blanco-Bercial et al. (2013) unpubl.	NWP2
*P. parvus*	KC287789	Japan Sea, Iki Island	Blanco-Bercial et al. (2013) unpubl.	NWP2
*P. parvus*	KC287798	Akkeshi Bay, Japan	Blanco-Bercial et al. (2013) unpubl.	NWP2
*P. parvus*	KC287799	Akkeshi Bay, Japan	Blanco-Bercial et al. (2013) unpubl.	NWP2
*P. parvus*	KC287800	Nakajima, Japan	Blanco-Bercial et al. (2013) unpubl.	NWP2
*P. parvus*	KC287801	Nakajima, Japan	Blanco-Bercial et al. (2013) unpubl.	NWP2
*P. parvus*	KC287788	Japan Sea, Iki Island	Blanco-Bercial et al. (2013) unpubl.	NWP3
*P. parvus*	KC287793	Akkeshi Bay, Japan	Blanco-Bercial et al. (2013) unpubl.	NWP4
*P. parvus*	KC287794	Nakajima	Blanco-Bercial et al. (2013) unpubl.	NWP5
*P. parvus*	KC287795	Nakajima	Blanco-Bercial et al. (2013) unpubl.	NWP6
*P. parvus*	EU856802	Chinese coastal waters	Sun S, Wang M and Li C (2008) unpubl.	NWP7
*P. parvus*	KC287797	Yellow Sea	Blanco-Bercial et al. (2013) unpubl.	NWP7
*P. parvus*	EU856803	Chinese coastal waters	Sun S, Wang M and Li C (2008) unpubl.	NWP8
*P. parvus*	EU856804	Chinese coastal waters	Sun S, Wang M and Li C (2008) unpubl.	NWP9
*P. parvus*	KC287785	Japan Sea, Iki island	Blanco-Bercial et al. (2013) unpubl.	NWP10
*P. parvus*	HM045398	Chinese coastal waters	Sun S, Wang M and Li C (2010) unpubl.	NWP11
*P. parvus*	EU599545	Chinese coastal waters	Sun S, Wang M and Liu B (2008) unpubl.	NWP12
*P. parvus*	EU856801	Chinese coastal waters	Sun S, Wang M and Liu B (2008) unpubl.	is *P. aculeatus*
*P. parvus*	HQ150069	Makassar Strait, Indonesia	Blanco-Bercial et al. (2011) [31]	PI2
*P. parvus*	AF474111	Off Okinawa	Bucklin A and Frost BW (2002) unpubl.	PI13
*P. parvus*	KC287782	off Okinawa	Blanco-Bercial et al. (2013) unpubl.	PI13
*P. indicus* s.l.	JQ911986	Tropical Pacific	Cornils and Blanco-Bercial (2013) [32]	PI14
*P. indicus* s.l.	JQ911985	Makassar Strait, Indonesia	Cornils and Blanco-Bercial (2013) [32]	PI15
*P. parvus*	KC287783	off Okinawa	Blanco-Bercial et al. (2013) unpubl.	PI16
*P. parvus*	KC287786	off Okinawa	Blanco-Bercial et al. (2013) unpubl.	PI17
*P. parvus*	JF905687	French Polynesia	Leray M, Agudelo N, Mills SC and Meyer CP (2011) unpubl.	PI18
*P. parvus*	KC594152	Kaneohe Bay, Oahu, Hawaii	Jungbluth and Lenz (2013) [33]	PI22
*P. parvus*	KC287790	off Okinawa	Blanco-Bercial et al. (2013) unpubl.	PT4
*P. quasimodo*	KC287771	Tunesia	Blanco-Bercial et al. (2013) unpubl.	PQ1
*P. quasimodo*	KC287805	NW Atlantic	Blanco-Bercial et al. (2013) unpubl.	PQ1
*P. quasimodo*	JQ911984	SW Medi-terranean Sea	Cornils and Blanco-Bercial (2013) [32]	PQ2
*P. quasimodo*	KC287772	Tunesia	Blanco-Bercial et al. (2013) unpubl.	PQ6
*P. indicus s.l.*	KC287773	Tunesia	Blanco-Bercial et al. (2013) unpubl.	PQ7
*P. quasimodo*	KC287775	Algeria	Blanco-Bercial et al. (2013) unpubl.	PQ8
*P. quasimodo*	KC287776	Algeria	Blanco-Bercial et al. (2013) unpubl.	PQ8
*P. indicus s.l.*	KC287777	Algeria	Blanco-Bercial et al. (2013) unpubl.	PQ9
*P. quasimodo*	KC287806	NW Atlantic	Blanco-Bercial et al. (2013) unpubl.	PQ10
*P. quasimodo*	KC287807	NW Atlantic	Blanco-Bercial et al. (2013) unpubl.	PQ11
*P. indicus s.l.*	KC287774	Y island,	Blanco-Bercial et al. (2013) unpubl.	SEI
		NW Australia		

Five species were identified combining the measurements of each individual prior to DNA extraction and the detailed morphological analysis of paratypes from each clade after the genetic analysis according to the morphological characteristics summarised by [34]. *Paracalanus nanus* can be distinguished from the other species by its small size and short antennules (barely reaching the end of the prosome), and the distal edges of the exopod segment 3 (Exp3) of the swimming legs 2 – 4 (P2-P4) are not serrated (Figure 1, Additional file 2). *Paracalanus tropicus* has very short urosome segments and as such a high P:U ratio. *Paracalanus indicus*, *Paracalanus parvus*, and *Paracalanus quasimodo* are distinguished by differences in the serration of the distal outer edge of the Exp3 of the P2-P4. *P. parvus* has a vaulted forehead, while a dorsalic hump on the prosome is present in *P. quasimodo. P. indicus* is characterised by posterior-dorsal spines on the female genital segment. Due to the process of net sampling, specimens were often lacking distal parts of the antennules and the swimming legs and, thus complicating the identification of the morphospecies.

### DNA extraction and amplification

DNA was extracted using the QIAamp DNA Mini Kit (Qiagen) from whole individuals and were eluted in 200 μl elution buffer (AE). DNA samples were stored at −20°C until further analysis. The mitochondrial protein coding genes COI and Cytb were sequenced using the primer sets UCYTB151F and UCYTB270R for Cytb [35], and LCO1490 and HCO2198 for COI [36], and the reverse COI primer C1-N-2191 (alias Nancy, 5`-CCCGGTAAAATTAAAATATAAACTTC-3`; [37]) for difficult specimens. PCR amplifications were performed in 25 μl reaction volumes. For Cyt *b* it included 5 μl 5x KAPA2G Buffer B, 5 μl 5x KAPA Enhancer 1, 0.125 μl of 100 μM each primer, 0.5 μl of 10 mM dNTPs, 0.15 μl of KAPA2G DNA Polymerase (5 Units/μl) and 2 μl DNA template. PCR reactions for Cytb consisted of 35 cycles of denaturation at 94°C for 40 sec, annealing at 50°C for 45 sec and extension at 72°C for 45 sec. For COI the reaction volume included 5 μl of 5x Colorless GoTaq® Flexi Buffer (Promega), 2.5 μl of 25 mM MgCl2, 0.1 μl of 100 μM each primer, 0.5 μl of 10 mM dNTPs, 0.13 μl GoTaq® Flexi DNA Polymerase (Promega) and 2 μl of DNA template. PCR reactions for COI consisted of 35 cycles of denaturation at 94°C for 40 sec, annealing at 45°C for 45 sec and extension at 69°C for 45 sec. PCR products were run on a 1% agarose/TBE gel and afterwards stained with ethidium bromide for band characterization. Positive results were purified with ExoSap-IT (0.25 μl exo, 1 μl SAP) and subsequently used for cycle sequencing with Big Dye Terminator Ver. 3.1 (Applied Biosystems Inc., ABI) and the same primers as for the PCR amplifications. The sequences were run on an ABI 3130xl DNA sequencer.

### Sequence editing

In CodonCode Aligner Vers. 3.7.1.1 (CodonCode Corporation) both strands were assembled into contigs, aligned and visually inspected for sequencing errors. 42 COI sequences named *Paracalanus parvus*, *Paracalanus indicus* or *Paracalanus quasimodo* from Genbank were included with the present data (Table 2). No additional COI sequences of other species from the *P. parvus* complex were found in GenBank (until June 30, 2013). One COI sequence from GenBank named *Paracalanus parvus* did not match with the other sequences of this species complex but showed close resemblance to *Paracalanus aculeatus* sequences [32]. It was excluded from the present analysis.

**Table 2:** Tests for species distinctivenes of the MOTUs for COI (165 sequences (including 43 GenBank sequences))

**MOTU**		**n**	**H**	**Contig size**	**Closest sister taxon**	**Intra uncorrected p-distance (MEGA)**		**Inter uncorrected p-distance (MEGA) to closest taxon**	
						**Min - Max**	**Mean**	**Min – Max**	**Mean**
PN	*P. nanus*	4	4	647	PI	0.002 – 0.028	0.018	0.117 – 0.144	0.128
PT	*P. tropicus*	5	5	647	PA	0.002 – 0.009	0.004	0.048 – 0.057	0.051
PA	Pan-Atlantic	17	5	641	PT	0.002 – 0.006	0.003	0.048 – 0.057	0.051
SEA/NZ	SE Atlantic/New Zealand	10	6	606	NWA	0.003 – 0.011	0.007	0.032 – 0.042	0.037
NWA	NW Atlantic	5	4	647	SEA	0.002 – 0.014	0.008	0.032 – 0.042	0.037
NEA	NE Atlantic	7	2	647	NWA	0.003	-	0.076 – 0.082	0.078
SWA	SW Atlantic	5	3	647	PT	0.003 – 0.006	0.005	0.142 – 0.147	0.144
SWP1	SW Pacific 1	1	1	647	SEI	-	-	0.124	-
SEI	SE Indic	1	1	600	SWP1	-	-	0.124	-
SWP	SW Pacific	5	2	647	NWP	0.019	-	0.122 – 0.130	0.125
NWP	NW Pacific	21	12	612	SWP	0.002 – 0.014	0.007	0.122 – 0.130	0.125
NEP	NE Pacific	6	3	647	NWP	0.002 – 0.009	0.006	0.114 - 0.125	0.120
SEP	SE Pacific	5	5	628	NEP	0.003 – 0.017	0.010	0.131 – 0.136	0.134
PQ	*P. quasimodo*	34	12	647	PI	0.002 – 0.016	0.006	0.081 – 0.106	0.094
PI	*P. indicus*	39	22	622	PQ	0.002 –0.034	0.014	0.081 – 0.106	0.094
Total		165	87						

n (specimen number), H (number of haplotypes).

Identical sequences were merged to unique haplotypes (Table 2) with Mesquite Ver. 2.75 [38]. Out of the 162 individuals analysed, 122 (COI) or 131 (Cytb) specimens could be extracted and amplified. The 165 sequences of COI (including 43 sequences from Genbank) could be placed in 87 haplotypes and the 131 Cytb sequences could be attributed to 49 haplotypes (Table 2, Additional file 3). Sequences are published in GenBank (KF715875 – KF715996; KF715999 – KF716129; Additional file 1). All COI sequences had a minimum length of 400 bp, and 91.2% had more than 600 bp.

### Tests for pseudogenes

Some COI sequences could not be sequenced due to double bands in the agarose gel. For a few other COI sequences, no consensus sequence could be built. Others produced highly divergent sequences (single sequences that differ extraordinarily from the alignment). These could be signs of either heteroplasmy, the presence of pseudogenes, contaminations, or the nonspecific binding of at least one primer under less stringent PCR conditions. All outlier sequences were excluded from the analyses. These difficulties were not found in Cytb. In the final alignments, no stop codons or indels (insertions-deletions) could be detected which would be indications for pseudogenes or incomplete lineage sorting [39,40]. The diversity for each codon position separately was also checked [41] using MEGA 5.2.2 [42]. In mitochondrial genes the diversity should be higher in the third codon position, while in pseudogenes the diversity would be equally distributed in all three codon positions. For COI the diversity varied between all three positions (1st position: 0.0579 ± 0.0091; 2nd position: 0.0006 ± 0.0002; 3rd position: 0.3319 ± 0.0136) and similar variations were found for Cytb (1st position: 0.0602 ± 0.0128; 2nd position: 0.0195 ± 0.0088; 3rd position: 0.3165 ± 0.0191). Furthermore, 392 of 647 positions of the COI data set and 214 of 351 positions of the Cytb data set were conserved. All these tests indicate the absence of pseudogenes in the final alignment. However, pseudogenes may be very similar to the targeted mitochondrial gene and thus be overlooked.

### Sequence analysis

The alignments of the haplotypes were used to infer Maximum Likelihood (ML) phylogenetic trees carried out with RAxML Vers. 7.2.8 [43] and Bayesian Inference (BI) calculated with MrBayes Vers. 3.2 [44]. The analyses were completed with un-partitioned nucleotides sequences, and with partitioned datasets accounting for the different substitution rates in the three codon positions. RAxML was run under the option GTRGAMMA and a complete random starting tree for the 10000 bootstrap replicates [45]. Then, a best-known likelihood tree search (500 inferences) was performed under GTRMIX and a completely random starting tree. The final tree topology was evaluated under GTRGAMMA to yield stable likelihood values. For the Bayesian Inference the nucleotide substitution model was selected with jModeltest Vers. 2.3 [46,47] using the Akaike information criterion (AIC) for small sample sizes. The best-fitted model (GTR + I + G) was then implemented in MrBayes. The analysis was run for 3,000,000 generations for the unpartitioned data set and for 10,000,000 generations for the codon model with a sample frequency of 1,000 generations. The first 500 trees were discarded as burn-in. Clade support is shown on the nodes of the trees as the Bayesian Posterior Probability (BPP) when BPP > 0.90.

As outgroup *Delibus* spp. from the family Paracalanidae were chosen (COI: Accession numbers JQ911978 (*Delibus nudus*); JQ911979, KF715873, KF715874 (*Delibus* sp.); Cytb: KF715997, KF715998 (*Delibus* sp.)). The analysis of the phylogeny of the family Paracalanidae has shown that the genus *Delibus* has the closest relationship to the *Paracalanus parvus* complex [32].

For presumably recently diverged lineages that were separated with some species delimitation methods haplotype networks were created with TCS Vers. 1.2.1 [48] to demonstrate the geographic structure of the haplotypes.

### Species delimitation methods

Two independent methods of species delimitation were applied to propose a first species hypothesis for the *Paracalanus parvus* complex: Automated Barcoding Gap Discovery (ABGD) and the generalized mixed Yule coalescent model (GMYC).

Automatic Barcode Gap Discovery (ABGD) is an automated iterative process to sort sequences into putative species based on pairwise distances without an *a priori* species hypothesis [17]. This algorithm automatically detects significant differences between intra- and inter-specific variations (i.e. barcoding gap). Aligned sequences of all haplotypes were uploaded to the web interface at http://wwwabi.snv.jussieu.fr/public/abgd/abgdweb.html and were run with the default settings. P (prior limit to intraspecific diversity) had a minimum value of 0.001 and a maximum value of 0.1. The relative gap width (X) had a value of 1. All available models (Jukes-Cantor (JC86), Kimura (K80)) were tested.

The generalized mixed Yule coalescent model (GMYC) infers species boundaries by measuring the transition from intra- to inter-species branching patterns [16]. This method combines models of stochastic lineage growth (Yule models) with coalescence theory. The analysis is implemented in R as part of the package “splits” (SPecies LImits by Threshold Statistics; [49]). The explanatory power of a model assuming a transition from population-specific to more phylogeny-like branching patterns is compared to a null model (all specimens are derived from a single species). The GMYC method was applied allowing a single threshold [50]. Prior to the analysis, outgroups were removed and the unique haplotypes were used to render an ultrametric consensus tree as starting point for the GMYC model (BEAST Vers. 1.7.4 [51]). A relaxed uncorrelated log-normal clock was chosen with a mean substitution rate fixed at 1 and estimated branch length with a coalescent prior. MCMC chains were run for 10 million generations sampling every 1,000 steps after a burn-in period of 1,000 trees.

As the both methods are sensitive to intra-species sampling we additionally analysed species delimitation for an alignment of all 165 specimens.

To test whether the MOTUs derived from the ABGD and GMYC analyses represent putative species, several species delimitation methods were applied. The resulting MOTUs were similar for the two gene fragments. Therefore, only the COI results are presented, since this gene fragment is frequently used to identify species (i.e. DNA barcoding; [52]), and distance thresholds can be compared to those of other copepods. Cytb results are found in the supplementary material. The methods all calculated species delimitation without *a priori* defined groups and are all based on uncorrected pairwise distance calculated with MEGA Vers. 5.2.2 unless otherwise indicated, since the use of K2P distance for DNA barcoding analysis is under debate [53]. Colour heat maps representing the distances between all haplotypes were plotted in MATLAB (base installation of R2013b, The MathWorks Inc.).

Barcode gaps between well-supported clades of haplotypes identified by Maximum Likelihood and Bayesian Inferences were taken as an indication of separate MOTUs. To find the optimal thresholds for intra-specific p-distances, the function localMinima of the SPIDER (SPecies IDentity and Evolution in R) Vers. 1.2 package for R (http://www.R-project.org) was used [54]. Based on the concept of the barcoding gap, this method indicates the transition between intra- and interspecific genetic distances from a dip in the density of the uncorrected p-distances without prior knowledge of species identity [54] and provides thresholds. These were used to cluster the sequences with the software jMOTU [15].

Rosenberg’s P(*AB*) examines whether monophyly has been produced by evolutionary processes or by insufficient sampling and calculates the probability that a MOTU with by *A* haplotypes is monophyletic to its closest relative with *B* haplotypes [18]. Significance of nodes was visualized with the R package SPIDER using a consensus ultrametric tree built in BEAST.

## Results

### Molecular species identification

**Figure 2:**
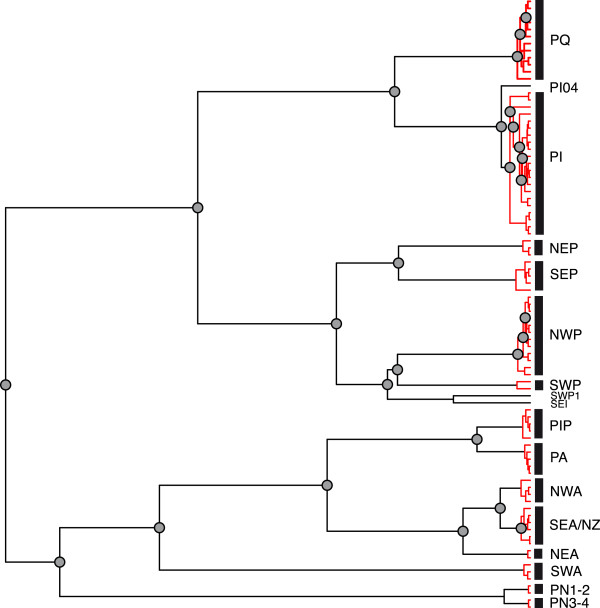
Results of GMYC (red lines) and Rosenberg (grey dots show separated nodes).

**Figure 3:**
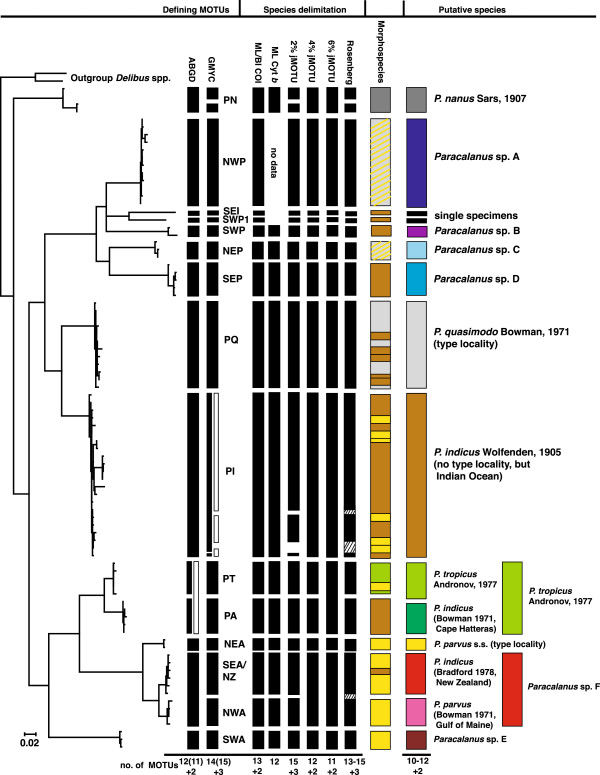
**Results of species delimitation methods (Maximum Likelihood analysis of Cytb (ML Cyt b), jMOTU analyses with 2, 4 and 6% thresholds, ABGD, GMYC and Rosenberg) with resulting putative species.** White columns in ABGD and GMYC reveal differences in species delimitation using an alignment with all 165 specimens instead of haplotypes (black columns). Colors of morphospecies match with the original described species name; transverse lines mark when identification and literature records disagree.

The primary species delimitation analysis based on 87 haplotypes with ABGD and GMYC resulted in 12 or 14 MOTUs, and two or three single sequences (Figures 2 and 3). The two methods were largely congruent in 10 MOTUs (NWP, SWP, NEP, SEP, PQ, PI, PT, PA, NEA, SWA). The two remaining MOTUs detected with ABGD were each divided in two groups with the GMYC analysis. One of the MOTUs (PN) included only individuals that were previously identified as *Paracalanus nanus*. This species was not the main target of this study and thus, we combined the two groups found in GMYC in one MOTU as suggested by ABGD analysis. The second MOTU included two groups separated in GMYC by their geographic distribution. These were counted as two separate MOTUs (SEA/NZ, NWA) to be tested with other methods. Thus, the species delimitation with these two methods resulted in 13 MOTUs, which were evaluated with other species delimitation methods. To test whether the use of haplotypes influenced the analysis of ABGD and GMYC we also analysed a data set with all 165 specimens. The resulting MOTUs of the two data sets were congruent with two exceptions. With the ABGD analysis the MOTUs PT and PA were fused, while with GMYC the MOTU PI was divided in three groups (Figure 3).

**Figure 4:**
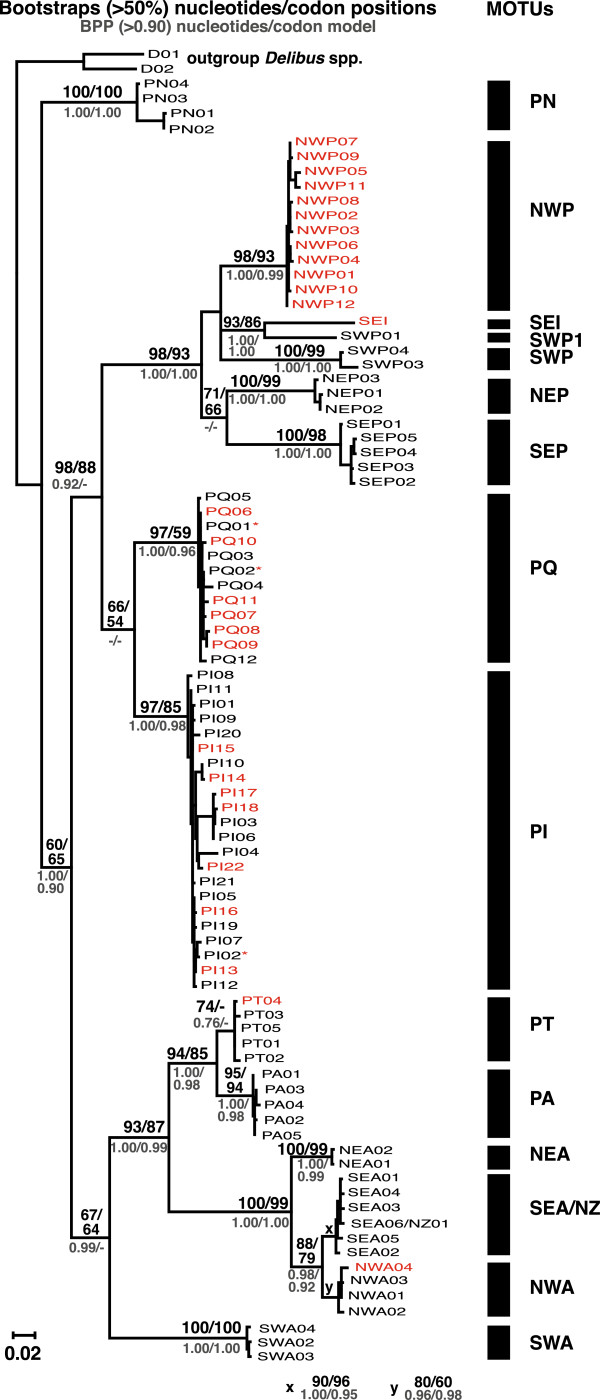
**RAxML Maximum Likelihood tree for haplotypes (COI).** Numbers show the percentage bootstrap support from two analyses: unmodified nucleotide sequences/ sequences separated by codon position, and bayesian posterior probability (BPP): unmodified nucleotide sequences/codon model. Haplotypes from GenBank are marked with red color, if only some sequences of a haplotype were taken from GenBank they are marked with *. Black bars display defined MOTUs.

The MOTUs were named either according to their geographic occurrence (NEA, SWA, SEA/NZ, NWA, NWP, NEP, SEP, SWP, SEI, PA) or for lineages with a wider spread distribution a potential species name, derived from morphological observations was used as abbreviation (PT, PQ, PI; Table 2). The MOTU NWP was exclusively built by sequences obtained from GenBank. Other sequences obtained from Genbank were placed in the MOTUs PQ, PI, PT and NWA (Table 1, Figures 3 and 4). These sequences could not be inspected for sequencing errors as no raw data were available.

The single sequences (SEI, SWP1, PI04) will not be considered as MOTUs at present until further sampling at their location (northern Australia) will either confirm or contradict their existence. The sequence PI04 had a p-distance of 2.8% to all other PI haplotypes and was thus singled out in GMYC.

The cladistic analyses of the COI haplotypes yielded 13 monophyletic clades and two single sequences (Figure 4), which were conform to the results from the ABGD and GMYC analyses. Twelve clades were well supported (>80% BS, >0.9 BPP) and one (PT) was moderately well supported (74% BS, <0.9 BPP). The cladistic analyses of Cytb haplotypes yielded twelve clades, identical to the clades for COI (Additional files 4 and 5). However, NWA and PI were not retrieved as monophyletic, but p-distances within these MOTUs were much lower than to their sister-taxon (Additional file 5). NWP and SEI were not found in the Cytb tree since they were based on GenBank sequences, and SWP1 could not be sequenced.

**Figure 5:**
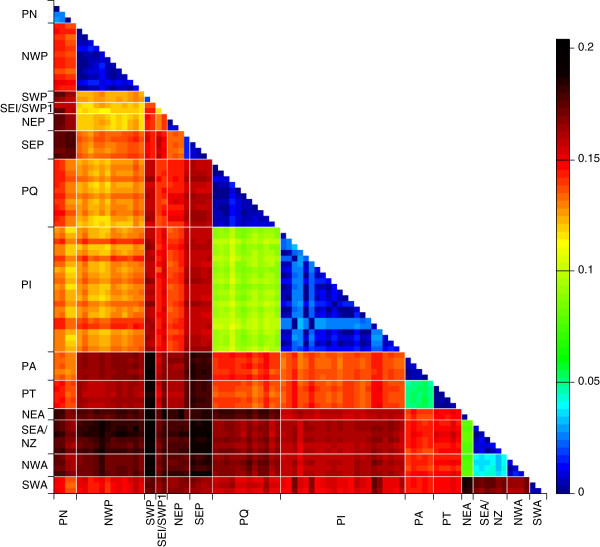
**Color heatmap representing uncorrected p-distances (COI) among the haplotypes of the *Paracalanus parvus *** **species complex.**

The mean uncorrected p-distances between MOTUs were generally higher than the divergence within the MOTUs (Table 2, Figure 5). Within MOTU sequence divergences varied between 0.2 (NEA) and 3.4% (PI), while differences between MOTUs varied between 3.2 – 14.8%. NEA shared haplotypes with eight recently published COI sequences from the North Sea and the Gullmarsfjord, Sweden [55]; GenBank Accession numbers: JX995215 - JX995222) reaching uncorrected p-distances of 0.6%.

Three thresholds between intra- and inter-specific distances were detected with the SPIDER package of R (0.0202 (2% jMOTU), 0.0441(4% jMOTU), 0.0648 (6%jMOTU)). These thresholds were used in jMOTU to separate clusters yielding between 11 (jMOTU, 6% pairwise intra-specific distance) and 15 MOTUs (jMOTU, 2% pairwise intra-specific distance). Two (SEI, SWP1) or three (SEI, SWP1, PI04) single sequences were found (Figure 3). At 2%jMOTU PN and PI were separated each in two groups. At 4%jMOTU and 6%MOTU SEA/NZ and NWA were fused and at 6%jMOTU also PT and PA. Rosenberg’s P(*AB*) showed significant nodes that would result in 13 to 15 MOTUs (Figure 3).

**Figure 6:**
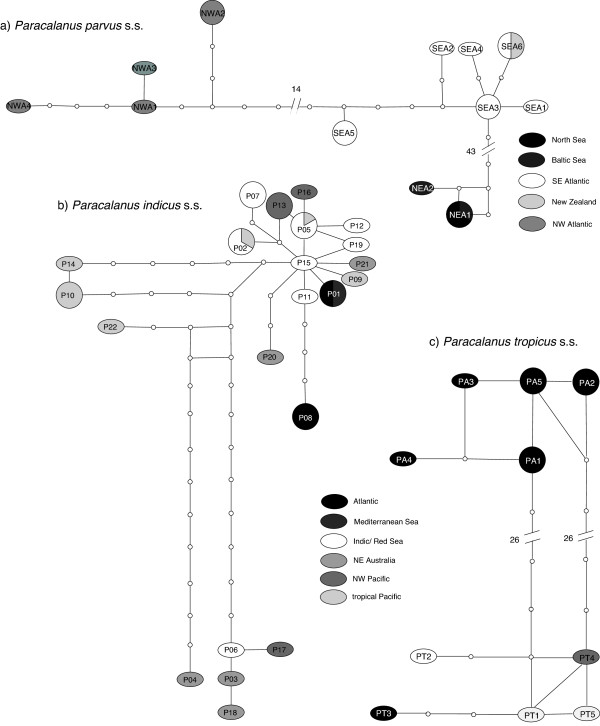
**TCS haplotype networks for recently evolved lineages in (a) *Paracalanus parvus *s.s., (b) *Paracalanus indicus *s.s. and (c) *Paracalanus tropicus *s.s..** Circles indicate haplotypes with more than one sequence.

All methods identified seven congruent MOTUs (NWP, SWP, NEP, SEP, PQ, NEA and SWA). PN and PI were in some analyses split into two or three groups (jMOTU 2%, GMYC, Rosenberg (Figures 2 and 3)). Four other PI haplotypes (PI03, PI06, PI17, PI18) were separated from PI in jMOTU 2% and significantly distinct from the other sequences in Rosenberg (Figures 2, 3 and 6). The heatmap and the haplotype network visualized close connections based on uncorrected p-distances between PT and PA, NEA, NWA and SEA/NZ, and PI and PQ (Figures 3 and 6). The latter were, however, separated in all methods. The MOTUs PT and PA were combined in jMOTU 6% (Figures 3 and 5). The MOTUs SEA/NZ and NWA showed the least divergence and were combined in jMOTU 4%, jMOTU 6%, and ABGD (Table 2, Figures 3 and 5).

### Phylogeography

**Figure 7:**
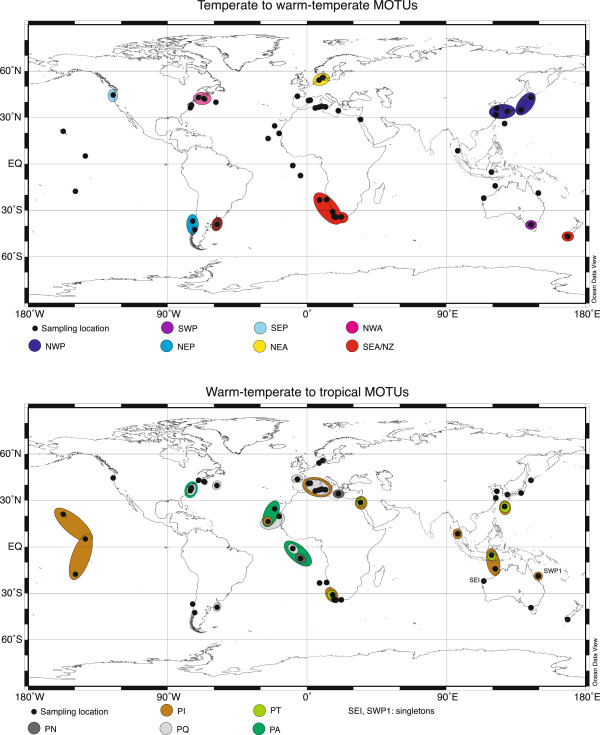
**Overview of sampling locations for specimens of the *Paracalanus parvus *complex (for exact information on latitude/longitude etc. see Additional file ****1****) and geographic distribution of MOTUs**.

Three categories of MOTUs were discovered regarding their geographic distribution (Figure 7): (1) Eight MOTUs occurred mainly in temperate waters and were restricted to one geographic region (NWA, NEA, SWA, NWP, NEP, SEP, SWP). Only SEA/NZ was found in two regions, Southeast Atlantic and Southwest Pacific waters, also sharing a haplotype (Figure 6). (2) Three MOTUs had a wider geographic distribution and some also occurred sympatrically. PQ seemed to be refined to the Atlantic and adjacent waters. PA was only retrieved from Atlantic Ocean samples while single PT specimens were found in the Indopacific, Red Sea and Southeast Atlantic. (3) PI was found in all oceans, mainly in the Indian and Pacific Ocean but also in the Atlantic Ocean and Mediterranean Sea. The two single sequences (SEI, SWP1) were found in the Northwest and Northeast off Australia.

Two locations for PN were also included in the analyses but we did not search particularly for this species on a global scale and thus its distribution cannot be evaluated here.

## Discussion

This study presents the first step to disentangling the genetic diversity of the ecologically important *Paracalanus parvus* species complex by using mitochondrial genes. Cleary this complex is composed of more MOTUs than morphologically described species and may thus be subject to cryptic and pseudocryptic speciation. The results provide a “global” framework for scientists identify individuals from the *Paracalanus parvus* complex according to their genetic affiliation (MOTU). It can also serve as a basis for future morphological taxonomy to test the validity of the found MOTUs.

### Genetic species delimitation

Independent methods without *a priori* defined groups are used to investigate the Paracalanus parvus complex (ABGD, GMYC, cladistic analyses (ML, BI), classical barcoding, Rosenberg’s P(AB)). These methods are generally congruent. They differ in the way that in some methods clades are subdivided or merged. In classical taxonomy this problem is well known and then nomenclature is helpful. In other words, one author defines two taxa as subspecies while another author defines the same taxa as separate species. It is important to note that the methods do not contradict each other in the subdivision but only in their assignment to hierarchy. Thus, the number of MOTUs varies between 11 and 15 MOTUs but not all of these may represent reproductively isolated species.

The seven MOTUs that are congruent in all analysis have genetic distances between MOTUS greater than 8% and therefore represent putative species. Speciation in marine copepods is assumed to have occurred when sequence divergences are approximately 8 – 9% (e.g. [56,57]. Some methods separate lineages within the level of intraspecific variety of copepods (1 – 4%, e.g. [56,58]. This accounts for the MOTUs SEA/NZ and NWA (uncorrected p-distance 3.2 – 4.2%) and for PA and PT (4.8 – 5.7% uncorrected p-distance). These two MOTUs may be recently diverged conspecific lineages, due to their geographic isolation, however, they show evidence of the possible existence of two species which would be in concordance with the unified species concept of [59]. In total, 10 to 12 putative species are found within the present genetic data set of the *Paracalanus parvus* species complex.

For this species complex, COI has provided a rapid and sufficient support for the evidence of cryptic and pseudocryptic speciation. The resulting putative species are often separated according to their geographic habitat, which provides additional support for the results of the species delimitation methods.

However, it is well known that single locus analysis of species delimitation may under- or overestimate the number of species due to e.g. pseudogenes, incomplete lineage sorting (e.g. [40]). An independent nuclear marker with a different level of gene flow will be needed in the future to validate the number of putative species found with a mitochondrial marker, which has been suggested by many authors e.g. [60,61]. The usage of a nuclear marker could also clarify whether the low genetic divergences between sister-lineages such as the geographically distinct NEA and SEA/NZ are a result from recent speciation events or from continuing gene flow between two populations. Thus, future studies should include not only more sampling locations and detailed morphological analysis, but also further molecular markers with an independent evolutionary history compared to mitochondrial genes and possibly interbreeding studies that could also help to distinguish between species. It has been shown that geographicallly isolated populations [62] or even groups with low COI sequence divergence [63] can be reproductively isolated.

### Molecular and morphological species identification

Morphological and molecular identification are not congruent. Morphospecies are found in more than one of the 10 to 12 putative species, except for *Paracalanus nanus*, which is conform with the MOTU PN and clearly identified by its small size and short antennules. In thoroughly revised calanoid genera such as *Clausocalanus* the morphological taxonomy is congruent with the molecular taxonomy [58]. Furthermore, for oncaeid copepods it has been shown that even the smallest morphological detail is significant in species identification [64]. An indication that this could also be important for *Paracalanus* species is that morphological variability within species has been noted previously (e.g. [24,34]; McKinnon (personal communication)). There is also evidence that speciation in copepods can occur without apparent morphological speciation (e.g. [65-67]) and morphological similarities may also emerge after genetic differentiation due to adaptation to a similar habitat (convergent evolution). Especially species in coastal systems are known to show strong genetic differentiation [68-70]. A thorough morphological revision of the taxon *Paracalanus* may reveal differences whether differences between MOTUs have been overlooked previously or whether genetic differentiation is due to behavioural adaptation [66].

### Putative species

Some NEA specimens are collected from the type locality of *P. parvus* (Helgoland, North Sea; [71]). They closely resemble the original description of Claus ([71]; vaulted forehead, lack of spinules on the posterior surfaces of the coxae of P2-P4) and are suggested to represent *P. parvus* s.s. (Figure 4). NWA and SEA/NZ populations are a sister-species to or a subspecies of P. parvus s.s. (NEA) but they are separated in all analysis. Hence, NWA and SEA/NZ are referred to as *Paracalanus* sp. F. Specimens from the Northwest Atlantic (NWA) and Southeast Atlantic (SEA) have been identified as *P. parvus* (e.g. [29,72]) and closely resemble *P. parvus* from Helgoland, while specimens from New Zealand have been described as *P. indicus*[34]. However, the specimens from New Zealand lack the typical postero-lateral spines on the genital segment and are also only little ornamented on the posterior surfaces of the swimming legs.

Specimens from the type locality of *Paracalanus indicus* (Maldive Islands, [73]) could not be obtained for the present study. However, specimens from the Andaman Sea (Indian Ocean) belonging to PI are morphologically congruent with the description of Wolfenden [73]. Hence, this MOTU is preliminary named *P. indicus*. Bowman [29] redescribed *P. indicus* from samples off Cape Hatteras (Northwest Atlantic, USA) but his drawings (Page 27 Figure twenty two c) show comparatively short urosomal segments 2 and 3 (resulting in a high P:U ratio). This is characteristic for *P. tropicus*[28], and present in specimens of PA and PT (Additional file 2). PA specimens are found near in the Northwest Atlantic near Cape Hatteras. These observations suggest that *P. indicus* described by Bowman [29] could be identical with PA. Due to low genetic divergence PA and PT are considered to be subspecies of *P. tropicus*.

*P. quasimodo* is distinguished from other species due to the presence of spinules at the distal outer edge of Exp3 of swimming leg four and many spinules on the posterior surfaces of the coxae of P2-P4. These characteristics are found in specimens of PQ, NWP, and NEP but only PQ is found near Cape Hatteras, the type locality of *P. quasimodo*[29]. Hence, it is suggested that PQ represents *P. quasimodo*.

Specimens from the locations of NWP, SEP, NEP, SWP and SWA have previously been named *P. parvus*, or *P. indicus* (e.g. [24,74,75]), although some have mentioned the morphological similarity of NWP and NEP to *P. quasimodo*[24,76]. They are probably not connected to any described species and are thus referred to as *Paracalanus* sp. A (NWP), *Paracalanus* sp. B (SWP), *Paracalanus* sp. C (NEP), *Paracalanus* sp. D (SEP) and *Paracalanus* sp. E (SWA).

### Phylogeography

Only *Paracalanus indicus* (PI) is truly widespread on an oceanic scale, sharing haplotypes between oceans. Gene flow seems to lack persistent geographic boundaries as has been seen in other copepod species (e.g. [77,78]. In the past, at least two morphological species (*P. parvus* and *P. indicus*) have been identified from many regions around the globe [72], but the present study revealed that *P. parvus* is restricted probably to the Northeast Atlantic. Hence, the findings of *P. parvus* around the oceans have to be distributed to other MOTUs.

Seven MOTUs are found in temperate waters, each being restricted to one marine temperate ecoregion as assigned by [79], with the exception of SEA/NZ. Temperate environments in the southern hemisphere were established with the onset of the Antarctic convergence (~23 MYA (millions years ago); [80]. With the closure of the Indonesian Seaway (~13 MYA), temperate marine environments developed also in the Northwest Pacific. Sequence divergences between widespread tropical and geographically restricted temperate MOTUs for both the Atlantic and the Pacific were similare (Pacific: 14.0% (PI/PQ vs. NWP/NEP/SEP/SWP) and Atlantic: 14.5% (PT/PA vs. NEA/SEA/NWA). However, there are no direct estimates of mutation rates for copepods, but molecular clock calibrations for COI for other crustaceans resulted in mutation rates of approximately 1.4% per million years [81]. Thus, the temperate and tropical clades could have diverged somewhere around 10 – 11 MYA, which would be after the closure of the Indonesian Seaways and the establishment of temperate habitats. But taking into account the substitution saturation often seen in mitochondrial DNA such as COI, these divergence times could be underestimated.

Due to closure of the Central American Seaway (~4.6 MYA; [82], the Gulf Stream intensified creating favourable habitats in the North Atlantic. Low pairwise distances (7.9%) between the populations of *Paracalanus* sp. F (NWA, SEA/NZ) and *P. parvus* s.s. (NEA) could indicate that speciation processes in the Atlantic may have occurred more recently than in the Pacific which would coincide with the timing of paleoceanographic changes described above. Thus, all *Paracalanus* species possibly have a tropical ancestor, and temperate forms may have evolved concurrent with paleoceanographic changes that led to the establishment of temperate marine environments. Due to their high abundances and consequently a high number of adaptive mutations *Paracalanus* may have a great potential to rapidly adapt genetically as has been hypothesized for oceanic zooplankton in general [83].

In some regions it has also been noted that *Paracalanus* is abundant only during certain seasons [84]. Thus, it can be speculated that ecological factors (such as seasonality, food sources) of the temperate *Paracalanus* species are another important issue that enhance speciation and also function as boundaries to gene flow. From *Acartia tonsa* it is known that different salinity regimes in close vicinity coincide with genetic divergence [69], while sea water temperature seems to control lineages of *Metridia lucens* in the Southern Atlantic [85]. It has also been suspected that planktonic taxa may drift anywhere but successfully reproduce only in their favourite environment [6]. Based on this assumption that sympatric or parapatric speciation processes may play a more important role in pelagic evolution than vicariant or allopatric models, which is supported by other findings [63]. However, the linages of *P. tropicus* may be subject to allopatric speciation as they are separated by ocean (PA in the Atlantic, PT in the Indo-Pacific). The genetic isolation between oceans is also found in other copepods species, e.g. *Clausocalanus lividus* and has been explained by the rising of the Isthmus of Panama [77].

SEA/NZ (*Paracalanus* sp. F) includes specimens from the Southeast Atlantic and New Zealand. Their presence in Southwest Pacific waters may be explained by two possibilities. The first hypothesis is that these specimens are transported regularly, possibly by the Antarctic Circumpolar Current, to southern New Zealand. Due to high gene flow they have not yet separated from the Southeast Atlantic specimens. This theory is supported by our observation that the sequenced specimens from New Zealand in this study are morphologically similar to the description of Bradford [34]. The second hypothesis is that individuals are transported to New Zealand in ballast water tanks of commercial ships. *P. parvus* s.l. is common in many coastal waters and often found in ballast water tanks (e.g. [86,87]). It has also been suggested that cosmopolitan distribution of many coastal species may be partly attributed to ballast water transport [88]. However, the present results indicate that both the circumglobal distribution of temperate coastal species and the influence on species distribution may be questionable in case of *Paracalanus*.

Furthermore, the presented biogeography of the *Paracalanus* species can be biased due to the low specimen number as has been shown for other marine organisms [4,88]. Small sample sizes and restricted geographic sampling suggested that the circumpolar crinoid species *Promachocrinus kerguelensis* as a complex of several cryptic species, some geographically limited and others widespread [4]. A later study included circum-Antarctic samples and revealed that all of the lineages were circumpolar [89]. This shows that there is a need of adequate geographic sampling. The present study includes samples from many locations but still there are large geographic gaps, which could either hide more cryptic species or change the present biogeography. The East Pacific is only covered by a few locations (coastal waters of Oregon and Chile). The same accounts for the Southwest Atlantic and the Indian Ocean.

## Conclusions

The circumglobal distribution of many marine planktonic copepod species is currently under debate (e.g. *Paracalanus parvus*, *Acartia tonsa*, *Oithona similis*, *Paracalanus parvus*). For the Paracalanus parvus species complex the present study has provided clear evidence for cryptic and pseudocryptic speciation, revealing 10 to 12 putative species with differing biogeographic distribution. All species delimitation methods were largely congruent, which indicates that the species diversity was effectively assessed. One major insight was that *Paracalanus parvus* s.s. was only identified from samples the northeastern Atlantic, and not panmictic.

COI has proven to be a good indicator of specimen identification in *Paracalanus* and thus the present data set can serve as a database for future identification of *Paracalanus* specimens from other locations.

In conclusion, the *Paracalanus parvus* species complex can serve as a role model to investigate cryptic speciation in other widely distributed marine copepod species complexes and may help to better understand speciation processes within the pelagic marine environment in the future.

## Competing interests

The authors declare that they have no competing interests.

## Authors’ contributions

AC and CH designed the general approach of this study. AC performed the analysis and wrote the manuscript. Both authors contributed in editing the manuscript.

## Supplementary Material

Additional file 1 Collection information and sampling locations for COI and Cytb. Click here for file

Additional file 2 **Morphological observations made prior to DNA extraction (A), and from paratype specimens (B) for each MOTU (molecular operational taxonomic unit).** Identification characters were selected according to Bradford [34], (all specimens except NWP in ethanol). For NWP only specimens in formalin were available. Abbreviations (see also Figure 1): n (Number of specimens); TL (Total length), SD (Standard deviation); P:U (Prosome:Urosome ratio); A1 (antennules); GS (genital segment); CR (Caudal rami); U3 (Urosome segment 3); U4 (Urosome segment 4); AS (Anal segment); P2, P3, P4 (swimming legs 2, 3, 4); Exp3 (Exopod segment 3); B1 (Coxa). Click here for file

Additional file 3 **Number of Cytb sequences (n), haplotypes (H) and contig sizes.** MOTU: Molecular operational taxonomic unit. Click here for file

Additional file 4 Results of Maximum Likelihood/Bayesian Inference analyses (ML/BI), jMOTU analyses with 3% and 5% thresholds (determined with the function localMinima of the R package spider), ABGD, GMYC and Rosenberg) with resulting putative species. Click here for file

Additional file 5 **Color heatmap representing uncorrected p-distances (Cyt *b*) among the haplotypes of the *Paracalanus parvus *** **species complex.** Click here for file
