# Supplementary material for: Evidence of cryptic and pseudocryptic speciation in the Paracalanus parvus species complex (Crustacea, Copepoda, Calanoida)
Source: Front Zool. 2014 Mar 2;11:19. doi: 10.1186/1742-9994-11-19 (PMC3948017; doi:10.1186/1742-9994-11-19)
Supplement: Additional file 1 — Collection information and sampling locations for COI and Cytb. [file 1742-9994-11-19-S1.docx]

**Additional File 1:** Collection information and sampling locations for COI and Cytochrom b (with GenBank Accession numbers)

| Specimen_ID | Collected_by | Collection_date | Country | Identified_by | Lat_Lon | Accession Number | |
| --- | --- | --- | --- | --- | --- | --- | --- |
|  |  |  |  |  |  | COI | Cytb |
| D0001 | Sigrid Schiel | 13-Nov-2007 | Atlantic Ocean: tropical eastern | Astrid Cornils | 1.0061 S 9.0017 W | KF715873 | KF715997 |
| D0002 | Sigrid Schiel | 13-Nov-2007 | Atlantic Ocean: tropical eastern | Astrid Cornils | 1.0061 S 9.0017 W | KF715874 | KF715998 |
| PN0001 | Sigrid Schiel | 15-Nov-2007 | Atlantic Ocean: tropical eastern | Astrid Cornils | 7.4356 S 6.1500 W | KF715940 | KF716029 |
| PN0002 | Sigrid Schiel | 15-Nov-2007 | Atlantic Ocean: tropical eastern | Astrid Cornils | 7.4356 S 6.1500 W | KF715941 | KF716030 |
| PN0003 | Ruth Böttger-Schnack | 24-Oct-2001 | Mediterranean Sea | Astrid Cornils | 34.4147 N 20.3286 E | KF715942 | KF716031 |
| PN0004 | Ruth Böttger-Schnack | 24-Oct-2001 | Mediterranean Sea | Astrid Cornils | 34.4147 N 20.3286 E | KF715943 | KF716032 |
| PN0005 | Ruth Böttger-Schnack | 24-Oct-2001 | Mediterranean Sea | Astrid Cornils | 34.4147 N 20.3286 E | - | KF716033 |
| PP0001 | William T. Peterson | 2011 | Pacific Ocean: Northeast, Oregon | Astrid Cornils | 44.6009 N 125.2441 W | KF715882 | KF716005 |
| PP0002 | William T. Peterson | 2011 | Pacific Ocean: Northeast, Oregon | Astrid Cornils | 44.6009 N 125.2441 W | KF715883 | KF716010 |
| PP0003 | William T. Peterson | 2011 | Pacific Ocean: Northeast, Oregon | Astrid Cornils | 44.6009 N 125.2441 W | KF715884 | KF716006 |
| PP0004 | William T. Peterson | 2011 | Pacific Ocean: Northeast, Oregon | Astrid Cornils | 44.6009 N 125.2441 W | KF715885 | KF716007 |
| PP0005 | William T. Peterson | 2011 | Pacific Ocean: Northeast, Oregon | Astrid Cornils | 44.6009 N 125.2441 W | KF715886 | KF716008 |
| PP0006 | William T. Peterson | 2011 | Pacific Ocean: Northeast, Oregon | Astrid Cornils | 44.6009 N 125.2441 W | KF715887 | KF716009 |
| PP0007 | Ann Bucklin | 20-Aug-2008 | Atlantic Ocean: Northwest | Astrid Cornils | 41.9000 N 65.9783 W | KF715888 | KF716011 |
| PP0008 | Ann Bucklin | 26-Aug-2008 | Gulf of Maine: Northwest Atlantic Ocean | Astrid Cornils | 43.1417 N 70.0117 W | KF715889 | KF716012 |
| PP0009 | Ann Bucklin | 20-Aug-2008 | Atlantic Ocean: Northwest | Astrid Cornils | 41.9000 N 65.9783 W | KF715890 | KF716013 |
| PP0010 | Ann Bucklin | 15-Aug-2008 | Atlantic Ocean: Northwest | Astrid Cornils | 38.2717 N 74.4067 W | KF715891 | KF716025 |
| PP0011 | Meriem Khelifi Touhami | 12-Jun-2006 | Mediterranean Sea: Algeria | Astrid Cornils | 36.8710 N 7.9000 E | KF715892 | KF716036 |
| PP0012 | Rainer Kiko | 30-Mar-2010 | Atlantic Ocean: Northwest | Astrid Cornils | 16.5400 N 25.0000 W | KF715948 | KF716079 |
| PP0013 | Hans Verheye | 14-Dec-2005 | Atlantic Ocean: Atlantic Ocean: South Africa | Astrid Cornils | 34.2289 S 18.0741 E | KF715893 | - |
| PP0014 | Hans Verheye | 14-Dec-2005 | Atlantic Ocean: Atlantic Ocean: South Africa | Astrid Cornils | 34.2289 S 18.0741 E | KF715894 | - |
| PP0015 | Lidia Yebra | 11-Jul-2011 | Spain: Mediterranean Sea, Algeciras | Astrid Cornils | 36.1769 N 5.4119 E | KF715895 | KF716037 |
| PP0016 | Ruth Böttger-Schnack | 22-Oct-2001 | Mediterranean Sea | Astrid Cornils | 36.9028 N 12.0028 E | KF715896 | KF716038 |
| PP0017 | Ann Bucklin | 23-Aug-2006 | Pacific Ocean: tropical | Astrid Cornils | 5.1831 N 143.0042 W | KF715897 | KF716049 |
| PP0018 | Ann Bucklin | 23-Aug-2006 | Pacific Ocean: tropical | Astrid Cornils | 5.1831 N 143.0042 W | KF715898 | KF716050 |
| PP0019 | Gertraud Schmidt | 23-Mar-2011 | Indian Ocean: Thailand, Similan Islands | Astrid Cornils | 8.6335 N 97.5366 E | KF715899 | KF716041 |
| PP0020 | Gertraud Schmidt | 23-Mar-2011 | Indian Ocean: Thailand, Similan Islands | Astrid Cornils | 8.6335 N 97.5366 E | KF715900 | KF716042 |
| PP0021 | Dave McKinnon |  | Indian Ocean: Scott Reef, Australia | Astrid Cornils | 14.0500 S 121.7667 E | KF715989 | - |
| PP0022 | Sigrid Schiel | 7-Mar-1999 | Gulf of Aqaba: Red Sea | Astrid Cornils | 28.8344 N 34.7325 E | KF715901 | KF716039 |
| PP0023 | Dave McKinnon |  | Coral Sea: Australia | Astrid Cornils | 18.6081 S 149.6448 E | KF715991 | - |
| PP0024 | Dave McKinnon |  | Coral Sea: Australia | Astrid Cornils | 18.6081 S 149.6448 E | KF715990 | - |
| PP0025 | Dave McKinnon |  | Coral Sea: Australia | Astrid Cornils | 18.6081 S 149.6448 E | KF715902 | KF716034 |
| PP0026 | Dave McKinnon |  | Coral Sea: Australia | Astrid Cornils | 18.6081 S 149.6448 E | KF715903 | - |
| PP0027 | Max Schulz | 15-Sep-2008 | Indonesia: SW Sulawesi | Astrid Cornils | 5.1250 S 119.3500 E | KF715904 | KF716043 |
| PP0028 | Max Schulz | 15-Sep-2008 | Indonesia: SW Sulawesi | Astrid Cornils | 5.1250 S 119.3500 E | KF715905 | - |
| PP0029 | Gertraud Schmidt | 23-Mar-2011 | Indian Ocean: Thailand, Similan Islands | Astrid Cornils | 8.6335 N 97.5366 E | KF715906 | KF716044 |
| PP0030 | Dave McKinnon |  | Indian Ocean: Scott Reef, Australia | Astrid Cornils | 14.0500 S 121.7667 E | KF715907 | - |
| PP0031 | Sigrid Schiel | 7-Mar-1999 | Gulf of Aqaba: Red Sea | Astrid Cornils | 28.8344 N 34.7325 E | KF715908 | - |
| PP0032 | Dave McKinnon |  | Indian Ocean: Scott Reef, Australia | Astrid Cornils | 14.0500 S 121.7667 E | KF715909 | - |
| PP0033 | Dave McKinnon |  | Indian Ocean: Scott Reef, Australia | Astrid Cornils | 14.0500 S 121.7667 E | KF715911 | KF716040 |
| PP0034 | Max Schulz | 15-Sep-2008 | Indonesia: SW Sulawesi | Astrid Cornils | 5.1250 S 119.3500 E | KF715912 | KF716045 |
| PP0035 | Max Schulz | 15-Sep-2008 | Indonesia: SW Sulawesi | Astrid Cornils | 5.1250 S 119.3500 E | KF715913 | KF716046 |
| PP0036 | Dave McKinnon |  | Indian Ocean: Scott Reef, Australia | Astrid Cornils | 14.0500 S 121.7667 E | KF715914 | KF716047 |
| PP0037 | Rainer Kiko | 30-Mar-2010 | Atlantic Ocean: Kapverde Islands | Astrid Cornils | 16.5400 N 25.0000 W | KF715915 | KF716048 |
| PP0038 | Rainer Kiko | 30-Mar-2010 | Atlantic Ocean: Kapverde Islands | Astrid Cornils | 16.5400 N 25.0000 W | KF715916 | - |
| PP0039 | Rainer Kiko | 30-Mar-2010 | Atlantic Ocean: Kapverde Islands | Astrid Cornils | 16.5400 N 25.0000 W | KF715917 | - |
| PP0040 | Ann Bucklin | 23-Aug-2006 | Pacific Ocean: tropical | Astrid Cornils | 5.1831 N 143.0042 W | KF715918 | - |
| PP0041 | Sigrid Schiel | 13-Nov-2007 | Atlantic Ocean: tropical eastern | Astrid Cornils | 1.0061 S 9.0017 W | KF715919 | KF716051 |
| PP0042 | Sigrid Schiel | 13-Nov-2007 | Atlantic Ocean: tropical eastern | Astrid Cornils | 1.0061 S 9.0017 W | KF715920 | KF716052 |
| PP0043 | Sigrid Schiel | 13-Nov-2007 | Atlantic Ocean: tropical eastern | Astrid Cornils | 1.0061 S 9.0017 W | KF715921 | KF716053 |
| PP0044 | Sigrid Schiel | 13-Nov-2007 | Atlantic Ocean: tropical eastern | Astrid Cornils | 1.0061 S 9.0017 W | KF715922 | KF716066 |
| PP0045 | Sigrid Schiel | 13-Nov-2007 | Atlantic Ocean: tropical eastern | Astrid Cornils | 1.0061 S 9.0017 W | KF715923 | KF716067 |
| PP0046 | Sigrid Schiel | 15-Nov-2007 | Atlantic Ocean: tropical eastern | Astrid Cornils | 7.4356 S 6.1500 W | KF715924 | KF716054 |
| PP0047 | Rainer Kiko | 30-Mar-2010 | Atlantic Ocean: Kapverde Islands | Astrid Cornils | 16.5400 N 25.0000 W | KF715925 | KF716055 |
| PP0048 | Rainer Kiko | 30-Mar-2010 | Atlantic Ocean: Kapverde Islands | Astrid Cornils | 16.5400 N 25.0000 W | KF715926 | KF716056 |
| PP0049 | Ann Bucklin | 16-Aug-2009 | Atlantic Ocean: Northwest | Astrid Cornils | 36.4367 N 75.2117 W | KF715927 | KF716057 |
| PP0050 | Sigrid Schiel | 13-Nov-2007 | Atlantic Ocean: tropical eastern | Astrid Cornils | 1.0061 S 9.0017 W | KF715928 | KF716058 |
| PP0051 | Sigrid Schiel | 15-Nov-2007 | Atlantic Ocean: tropical eastern | Astrid Cornils | 7.4356 S 6.1500 W | KF715929 | KF716059 |
| PP0052 | Sigrid Schiel | 15-Nov-2007 | Atlantic Ocean: tropical eastern | Astrid Cornils | 7.4356 S 6.1500 W | KF715930 | KF716068 |
| PP0053 | Sigrid Schiel | 5-Nov-2007 | Atlantic Ocean: tropical eastern | Astrid Cornils | 24.6747 N 20.7511 W | KF715931 | KF716060 |
| PP0054 | Sigrid Schiel | 5-Nov-2007 | Atlantic Ocean: tropical eastern | Astrid Cornils | 24.6747 N 20.7511 W | KF715932 | KF716061 |
| PP0055 | Sigrid Schiel | 5-Nov-2007 | Atlantic Ocean: tropical eastern | Astrid Cornils | 24.6747 N 20.7511 W | KF715933 | KF716062 |
| PP0056 | Rainer Kiko | 30-Mar-2010 | Atlantic Ocean: Kapverde Islands | Astrid Cornils | 16.5400 N 25.0000 W | KF715934 | KF716063 |
| PP0057 | Sigrid Schiel | 13-Nov-2007 | Atlantic Ocean: tropical eastern | Astrid Cornils | 1.0061 S 9.0017 W | KF715935 | KF716064 |
| PP0058 | Sigrid Schiel | 7-Mar-1999 | Gulf of Aqaba: Red Sea | Astrid Cornils | 28.8344 N 34.7325 E | KF715936 | KF716069 |
| PP0059 | Max Schulz | 15-Sep-2008 | Indonesia: SW Sulawesi | Astrid Cornils | 5.1250 S 119.3500 E | KF715937 | KF716070 |
| PP0060 | Hans Verheye | 14-Dec-2005 | Atlantic Ocean: South Africa | Astrid Cornils | 34.2289 S 18.0741 E | KF715938 | KF716071 |
| PP0061 | Ben Kurten | 12-Apr-2012 | Indian Ocean: Red Sea | Astrid Cornils | 19.6081 N 38.7547 E | KF715939 | - |
| PP0062 | Ilka Peeken | 30-Jul-2006 | Mauritania: Atlantic Ocean | Astrid Cornils | 19.8333 N 6.1500 W | KF715944 | - |
| PP0063 | Sigrid Schiel | 13-Nov-2007 | Atlantic Ocean: tropical eastern | Astrid Cornils | 1.0061 S 9.0017 W | KF715945 | KF716091 |
| PP0064 | Lidia Yebra | 13-Aug-2011 | Spain: Mediterranean Sea, Lobregat | Astrid Cornils | 41.2897 N 2.1286 E | KF715946 | KF716092 |
| PP0065 | Lidia Yebra | 13-Aug-2011 | Spain: Mediterranean Sea, Lobregat | Astrid Cornils | 41.2897 N 2.1286 E | KF715947 | KF716093 |
| PP0066 | Meriem Khelifi Touhami | 12-Jun-2006 | Mediterranean Sea: Algeria | Astrid Cornils | 36.8710 N 7.9000 E | KF715949 | KF716080 |
| PP0067 | Leocadio Blanco-Bercial | 26-Jun-1905 | Spain: Bay of Biscay | Astrid Cornils | 43.7000 N 6.1500 W | KF715950 | KF716081 |
| PP0068 | Rainer Kiko | 30-Mar-2010 | Atlantic Ocean: Kapverde Islands | Astrid Cornils | 16.5400 N 25.0000 W | KF715951 | KF716082 |
| PP0069 | Rainer Kiko | 30-Mar-2010 | Atlantic Ocean: Kapverde Islands | Astrid Cornils | 16.5400 N 25.0000 W | KF715910 | - |
| PP0070 | Ilka Peeken | 30-Jul-2006 | Mauritania: Atlantic Ocean | Astrid Cornils | 19.8333 N 6.1500 W | KF715952 | KF716083 |
| PP0071 | Ilka Peeken | 30-Jul-2006 | Mauritania: Atlantic Ocean | Astrid Cornils | 19.8333 N 6.1500 W | KF715953 | - |
| PP0072 | Sigrid Schiel | 13-Nov-2007 | Atlantic Ocean: tropical eastern | Astrid Cornils | 1.0061 S 9.0017 W | KF715954 | KF716084 |
| PP0073 | Leocadio Blanco-Bercial | 1-Jan-2004 | Spain: Bay of Biscay | Astrid Cornils | 43.7000 N 6.1500 W | KF715955 | - |
| PP0074 | Ruth Böttger-Schnack | 22-Oct-2001 | Mediterranean Sea | Astrid Cornils | 36.9028 N 12.0028 E | KF715956 | KF716085 |
| PP0075 | Ruth Böttger-Schnack | 20-Oct-2001 | Mediterranean Sea | Astrid Cornils | 37.9975 N 5.9989 E | KF715957 | KF716086 |
| PP0076 | Meriem Khelifi Touhami | 12-Jun-2006 | Mediterranean Sea: Algeria | Astrid Cornils | 36.8710 N 7.9000 E | KF715958 | KF716090 |
| PP0077 | Ann Bucklin | 16-Aug-2009 | Atlantic Ocean: Northwest | Astrid Cornils | 36.4367 N 75.2117 W | KF715959 | KF716072 |
| PP0078 | Ann Bucklin | 16-Aug-2009 | Atlantic Ocean: Northwest | Astrid Cornils | 36.4367 N 75.2117 W | KF715960 | KF716073 |
| PP0079 | Ann Bucklin | 16-Aug-2009 | Atlantic Ocean: Northwest | Astrid Cornils | 36.4367 N 75.2117 W | KF715961 | KF716074 |
| PP0080 | Ilka Peeken | 30-Jul-2006 | Mauritania: Atlantic Ocean | Astrid Cornils | 19.8333 N 6.1500 W | KF715962 | KF716087 |
| PP0081 | Ilka Peeken | 30-Jul-2006 | Mauritania: Atlantic Ocean | Astrid Cornils | 19.8333 N 6.1500 W | KF715963 | - |
| PP0082 | Sigrid Schiel | 13-Nov-2007 | Atlantic Ocean: tropical eastern | Astrid Cornils | 1.0061 S 9.0017 W | KF715964 | KF716075 |
| PP0083 | Sigrid Schiel | 20-Nov-2007 | Atlantic Ocean: tropical eastern | Astrid Cornils | 23.2383 S 8.2383 E | KF715965 | - |
| PP0084 | Ann Bucklin | 16-Aug-2009 | Atlantic Ocean: Northwest | Astrid Cornils | 36.4367 N 75.2117 W | KF715966 | KF716094 |
| PP0085 | Monica Hoffmeyer | 13-Apr-2012 | Atlantic Ocean: Argentina, Mar del Plata | Astrid Cornils | 38.8914 S 57.9003 W | KF715982 | KF716124 |
| PP0086 | Sigrid Schiel | 20-Nov-2007 | Atlantic Ocean: tropical eastern | Astrid Cornils | 23.2383 S 8.2383 E | KF715967 | KF716096 |
| PP0087 | Sigrid Schiel | 20-Nov-2007 | Atlantic Ocean: tropical eastern | Astrid Cornils | 23.2383 S 8.2383 E | KF715968 | KF716103 |
| PP0088 | Holger Auel | 29-Jan-2004 | Atlantic Ocean: Namibia | Astrid Cornils | 22.8032 S 12.8101 E | KF715969 | KF716097 |
| PP0089 | Hans Verheye | 25-Oct-2006 | Atlantic Ocean: South Africa | Astrid Cornils | 34.0833 S 18.4167 E | KF715970 | KF716107 |
| PP0090 | Hans Verheye | 18-Oct-2006 | Atlantic Ocean: South Africa | Astrid Cornils | 30.8333 S 16.6667 E | KF715971 | KF716108 |
| PP0091 | Hans Verheye | 23-Oct-2006 | Atlantic Ocean: South Africa | Astrid Cornils | 33.8333 S 17.6667 E | KF715972 | KF716109 |
| PP0092 | Hans Verheye | 25-Oct-2006 | Atlantic Ocean: South Africa | Astrid Cornils | 34.0833 S 18.4167 E | KF715973 | KF716114 |
| PP0093 | Hans Verheye | 18-Oct-2006 | Atlantic Ocean: South Africa | Astrid Cornils | 30.8333 S 16.6667 E | KF715974 | KF716115 |
| PP0094 | Janet Grieve |  | New Zealand: Foveaux Strait | Astrid Cornils | 46.9403 S 168.4863 E | KF715975 | KF716098 |
| PP0095 | Hans Verheye | 16-Nov-2005 | Atlantic Ocean: South Africa | Astrid Cornils | 34.1500 S 22.3333 E | KF715976 | KF716110 |
| PP0096 | Ruben Escribano |  | Chile | Astrid Cornils | 36.8825 S 73.7622 W | KF715977 | - |
| PP0097 | Ruben Escribano |  | Chile | Astrid Cornils | 36.8825 S 73.7622 W | KF715978 | - |
| PP0098 | Carin Jantzen | 28-Feb-2011 | Chile: Comau fjord | Astrid Cornils | 42.3792 S 72.4438 W | KF715979 | KF716118 |
| PP0099 | Carin Jantzen | 28-Feb-2011 | Chile: Comau fjord | Astrid Cornils | 42.3792 S 72.4438 W | KF715980 | KF716119 |
| PP0100 | Carin Jantzen | 28-Feb-2011 | Chile: Comau fjord | Astrid Cornils | 42.3792 S 72.4438 W | KF715981 | KF716120 |
| PP0101 | Dave McKinnon |  | Coral Sea: Australia | Astrid Cornils | 18.6081 S 149.6448 E | KF715988 | - |
| PP0102 | Dave McKinnon | 2012 | Pacific Ocean: Australia, Melbourne | Astrid Cornils | 44.6009 N 125.2441 W | KF715992 | - |
| PP0103 | Dave McKinnon | 2012 | Pacific Ocean: Australia, Melbourne | Astrid Cornils | 44.6009 N 125.2441 W | KF715993 | - |
| PP0104 | Dave McKinnon | 2012 | Pacific Ocean: Australia, Melbourne | Astrid Cornils | 44.6009 N 125.2441 W | KF715994 | KF716121 |
| PP0105 | Dave McKinnon | 2012 | Pacific Ocean: Australia, Melbourne | Astrid Cornils | 44.6009 N 125.2441 W | KF715995 | KF716123 |
| PP0106 | Dave McKinnon | 2012 | Pacific Ocean: Australia, Melbourne | Astrid Cornils | 44.6009 N 125.2441 W | KF715996 | KF716122 |
| PP0107 | Astrid Cornils | 10-Oct-2010 | North Sea: Helgoland | Astrid Cornils | 54.1883 N 7.9000 E | - | KF715999 |
| PP0108 | Astrid Cornils | 10-Oct-2010 | North Sea: Helgoland | Astrid Cornils | 54.1883 N 7.9000 E | KF715876 | - |
| PP0109 | Astrid Cornils | 10-Oct-2010 | North Sea: Helgoland | Astrid Cornils | 54.1883 N 7.9000 E | KF715877 | - |
| PP0110 | Astrid Cornils | 10-Oct-2010 | North Sea: Helgoland | Astrid Cornils | 54.1883 N 7.9000 E | KF715878 | - |
| PP0111 | Sigrid Schiel | 17-Sep-2010 | Internal Denmark waters: Baltic Sea, Kattegat | Astrid Cornils | 55.8656 N 10.3793 E | KF715879 | - |
| PP0112 | Sigrid Schiel | 17-Sep-2010 | Internal Denmark waters: Baltic Sea, Kattegat | Astrid Cornils | 55.8656 N 10.3793 E | KF715880 | - |
| PP0113 | Sigrid Schiel | 17-Sep-2010 | Internal Denmark waters: Baltic Sea, Kattegat | Astrid Cornils | 55.8656 N 10.3793 E | KF715881 | - |
| PP0114 | Monica Hoffmeyer | 13-Apr-2012 | Atlantic Ocean: Argentina, Mar del Plata | Astrid Cornils | 38.8914 S 57.9003 W | KF715983 | KF716125 |
| PP0115 | Monica Hoffmeyer | 13-Apr-2012 | Atlantic Ocean: Argentina, Mar del Plata | Astrid Cornils | 38.8914 S 57.9003 W | KF715984 | KF716126 |
| PP0116 | Monica Hoffmeyer | 13-Apr-2012 | Atlantic Ocean: Argentina, Mar del Plata | Astrid Cornils | 38.8914 S 57.9003 W | KF715985 | KF716127 |
| PP0117 | Monica Hoffmeyer | 13-Apr-2012 | Atlantic Ocean: Argentina, Mar del Plata | Astrid Cornils | 38.8914 S 57.9003 W | KF715986 | KF716129 |
| PP0118 | Monica Hoffmeyer | 13-Apr-2012 | Atlantic Ocean: Argentina, Mar del Plata | Astrid Cornils | 38.8914 S 57.9003 W | KF715987 | KF716128 |
| PP0119 | Astrid Cornils | 10-Oct-2010 | North Sea: Helgoland | Astrid Cornils | 54.1883 N 7.9000 E | - | KF716002 |
| PP0120 | Astrid Cornils | 10-Oct-2010 | North Sea: Helgoland | Astrid Cornils | 54.1883 N 7.9000 E | - | KF716003 |
| PP0121 | Sigrid Schiel | 17-Sep-2010 | Internal Denmark waters: Baltic Sea, Kattegat | Astrid Cornils | 55.8656 N 10.3793 E | - | KF716004 |
| PP0122 | Sigrid Schiel | 17-Sep-2010 | Internal Denmark waters: Baltic Sea, Kattegat | Astrid Cornils | 55.8656 N 10.3793 E | - | KF716000 |
| PP0123 | Sigrid Schiel | 17-Sep-2010 | Internal Denmark waters: Baltic Sea, Kattegat | Astrid Cornils | 55.8656 N 10.3793 E | - | KF716001 |
| PP0124 | Leocadio Blanco-Bercial | 1-Jan-2004 | Spain: Bay of Biscay | Astrid Cornils | 43.7000 N 6.1500 W | - | KF716076 |
| PP0125 | Leocadio Blanco-Bercial | 1-Jan-2004 | Spain: Bay of Biscay | Astrid Cornils | 43.7000 N 6.1500 W | - | KF716077 |
| PP0126 | Ann Bucklin | 26-Aug-2008 | Gulf of Maine: Northwest Atlantic Ocean | Astrid Cornils | 43.1417 N 70.0117 W | - | KF716014 |
| PP0127 | Ann Bucklin | 26-Aug-2008 | Gulf of Maine: Northwest Atlantic Ocean | Astrid Cornils | 43.1417 N 70.0117 W | - | KF716015 |
| PP0128 | Ann Bucklin | 26-Aug-2008 | Gulf of Maine: Northwest Atlantic Ocean | Astrid Cornils | 43.1417 N 70.0117 W | - | KF716026 |
| PP0129 | Ann Bucklin | 26-Aug-2008 | Gulf of Maine: Northwest Atlantic Ocean | Astrid Cornils | 43.1417 N 70.0117 W | - | KF716016 |
| PP0130 | Ann Bucklin | 26-Aug-2008 | Gulf of Maine: Northwest Atlantic Ocean | Astrid Cornils | 43.1417 N 70.0117 W | - | KF716017 |
| PP0131 | Sigrid Schiel | 20-Nov-2007 | Atlantic Ocean: tropical eastern | Astrid Cornils | 23.2383 S 8.2383 E | - | KF716099 |
| PP0132 | Sigrid Schiel | 20-Nov-2007 | Atlantic Ocean: tropical eastern | Astrid Cornils | 23.2383 S 8.2383 E | - | KF716100 |
| PP0133 | Hans Verheye | 14-Dec-2005 | Atlantic Ocean: South Africa | Astrid Cornils | 34.2289 S 18.0742 E | - | KF716104 |
| PP0134 | Sigrid Schiel | 13-Nov-2007 | Atlantic Ocean: tropical eastern | Astrid Cornils | 1.0061 S 9.0017 W | - | KF716065 |
| PP0135 | Ann Bucklin | 15-Aug-2008 | Atlantic Ocean: Northwest | Astrid Cornils | 38.2717 N 74.4067 W | - | KF716018 |
| PP0136 | Ann Bucklin | 16-Aug-2009 | Atlantic Ocean: Northwest | Astrid Cornils | 36.4367 N 75.2117 W | - | KF716019 |
| PP0137 | Holger Auel | 29-Jan-2004 | Atlantic Ocean: Namibia | Astrid Cornils | 22.8032 S 12.8101 E | - | KF716105 |
| PP0138 | Holger Auel | 29-Jan-2004 | Atlantic Ocean: Namibia | Astrid Cornils | 22.8032 S 12.8101 E | - | KF716020 |
| PP0139 | Holger Auel | 29-Jan-2004 | Atlantic Ocean: Namibia | Astrid Cornils | 22.8032 S 12.8101 E | - | KF716111 |
| PP0140 | Holger Auel | 29-Jan-2004 | Atlantic Ocean: Namibia | Astrid Cornils | 22.8032 S 12.8101 E | - | KF716106 |
| PP0141 | Holger Auel | 29-Jan-2004 | Atlantic Ocean: Namibia | Astrid Cornils | 22.8032 S 12.8101 E | - | KF716113 |
| PP0142 | Ann Bucklin | 15-Aug-2008 | Atlantic Ocean: Northwest | Astrid Cornils | 37.6906 N 74.5117 W | - | KF716021 |
| PP0143 | Ann Bucklin | 15-Aug-2008 | Atlantic Ocean: Northwest | Astrid Cornils | 37.6906 N 74.5117 W | - | KF716027 |
| PP0144 | Ann Bucklin | 15-Aug-2008 | Atlantic Ocean: Northwest | Astrid Cornils | 37.6906 N 74.5117 W | - | KF716022 |
| PP0145 | Ann Bucklin | 15-Aug-2008 | Atlantic Ocean: Northwest | Astrid Cornils | 37.6906 N 74.5117 W | - | KF716028 |
| PP0146 | Ann Bucklin | 20-Aug-2008 | Atlantic Ocean: Northwest | Astrid Cornils | 41.9000 N 65.9783 W | - | KF716023 |
| PP0147 | Ann Bucklin | 20-Aug-2008 | Atlantic Ocean: Northwest | Astrid Cornils | 41.9000 N 65.9783 W | - | KF716024 |
| PP0148 | Janet Grieve |  | New Zealand: Foveaux Strait | Astrid Cornils | 46.9403 S 168.4863 E | - | KF716101 |
| PP0149 | Janet Grieve |  | New Zealand: Foveaux Strait | Astrid Cornils | 46.9403 S 168.4863 E | - | KF716102 |
| PP0150 | Hans Verheye | 23-Oct-2006 | Atlantic Ocean: South Africa | Astrid Cornils | 33.8333 S 17.6667 E | - | KF716112 |
| PP0151 | Lidia Yebra | 13-Aug-2011 | Spain: Mediterranean Sea, Lobregat | Astrid Cornils | 41.2897 N 2.1286 E | - | KF716095 |
| PP0152 | Lidia Yebra | 11-Jul-2011 | Spain: Mediterranean Sea, Algeciras | Astrid Cornils | 36.1769 N 5.4119 E | - | KF716035 |
| PP0153 | Lidia Yebra | 11-Jul-2011 | Spain: Mediterranean Sea, Algeciras | Astrid Cornils | 36.1769 N 5.4119 E | - | KF716078 |
| PP0154 | Lidia Yebra | 11-Aug-2011 | Spain: Mediterranean Sea, Tarragona | Astrid Cornils | 41.0725 N 1.1939 E | - | KF716088 |
| PP0155 | Lidia Yebra | 11-Aug-2011 | Spain: Mediterranean Sea, Tarragona | Astrid Cornils | 41.0725 N 1.1939 E | - | KF716089 |
| PP0156 | Hans Verheye | 16-Nov-2005 | Atlantic Ocean: South Africa | Astrid Cornils | 34.1500 S 22.3333 E | - | KF716116 |
| PP0157 | Hans Verheye | 16-Nov-2005 | Atlantic Ocean: South Africa | Astrid Cornils | 34.1500 S 22.3333 E | - | KF716117 |
