# Supplementary material for: Evidence of cryptic and pseudocryptic speciation in the Paracalanus parvus species complex (Crustacea, Copepoda, Calanoida)
Source: Front Zool. 2014 Mar 2;11:19. doi: 10.1186/1742-9994-11-19 (PMC3948017; doi:10.1186/1742-9994-11-19)
Supplement: Additional file 2 — Morphological observations made prior to DNA extraction (A), and from paratype specimens (B) for each MOTU (molecular operational taxonomic unit). Identification characters were selected according to Bradford [34], (all specimens except NWP in ethanol). For NWP only specimens in formalin were available. Abbreviations (see also Figure 1): n (Number of specimens); TL (Total length), SD (Standard deviation); P:U (Prosome:Urosome ratio); A1 (antennules); GS (genital segment); CR (Caudal rami); U3 (Urosome segment 3); U4 (Urosome segment 4); AS (Anal segment); P2, P3, P4 (swimming legs 2, 3, 4); Exp3 (Exopod segment 3); B1 (Coxa). [file 1742-9994-11-19-S2.docx]

Supplementary Table 2: Morphological observations made prior to DNA extraction (**A**), and from paratype specimens (**B**) for each MOTU (molecular operational taxonomic unit). Identification characters were selected according to Bradford [31], (all specimens except NWP in ethanol). For NWP only specimens in formalin were available. Abbreviations (see also Figure 1): n (Number of specimens); TL (Total length), SD (Standard deviation); P:U (Prosome:Urosome ratio); A1 (antennules); GS (genital segment); CR (Caudal rami); U3 (Urosome segment 3); U4 (Urosome segment 4); AS (Anal segment); P2, P3, P4 (swimming legs 2, 3, 4); Exp3 (Exopod segment 3); B1 (Coxa).

|  | **A** |  |  |  |  |  |  | **B** |  |  |  |  |  |
| --- | --- | --- | --- | --- | --- | --- | --- | --- | --- | --- | --- | --- | --- |
| MOTU | n | TL(µm) | SD | P:U | SD | shape of forehead | length of A1 relative to body segments | Serration of distal outer edge Exp3 (P2/P3/P4) | B1 wirh many posterior surface spinules (P2-P4) | Genital segmetn with posterior spinules | n | Morphotype | Remarks |
| PN | 5 | 623 | 28.5 | 3.09 | 0.23 | not vaulted | GS | yes/yes/yes | no | no | 3 | *P. nanus* |  |
| PT | 3 | 671 | 7.2 | 3.95 | 0.69 | not vaulted | CR | yes/yes/no | yes | ? | 3 | *P. tropicus* |  |
| PA | 18 | 747 | 33.2 | 3.56 | 0.29 | not vaulted | U3 | yes/yes/no | yes | yes | 4 | *P. indicus* |  |
| SEA/NZ | 27 | 909 | 82.5 | 3.29 | 0.35 | vaulted | AS/CR | yes/yes/no | no (only little) | no | 4 | *P. parvus* | in NZ *P. indicus* |
| NWA | 14 | 891 | 58.4 | 3.36 | 0.30 | vaulted | AS/CR | yes/yes?/no  (very small) | no | no | 2 | *P. parvus* |  |
| NEA | 12 | 892 | 63.1 | 3.18 | 0.15 | vaulted | U4/AS | Yes/ Yes (NorthSEa), Batic No/no | no | no | 9 | *P. parvus* | Baltic Sea smaller |
| SWA | 5 | 768 | 32.6 | 3.04 | 0.15 | not vaulted | AS | ? | no | no | 3 | ? | in references *P. parvus* |
| SWP1 | 1 | 738 | - | 3.21 | - | ? | ? | only a single sequence, no paratypes found | | | | | |
| SEI | sequence from GenBank | | | | | | |  |  |  |  |  |  |
| SWP | 5 | 675 | 53.8 | 2.93 | 0.21 | ? | AS/CR | yes/yes/no | ? | ? | 3 | *P. indicus* | Vial 435 |
| NWP (only paratypes) | 5 | 920 | 45.6 | 3.44 | 0.23 | humped | U4/AS | yes/yes/?  (broken legs) | yes (many) | no | 4 | *P. quasimodo* strongly ornamented | in formalin |
| NEP | 9 | 865 | 35.8 | 3.17 | 0.46 | humped | AS/CR | yes/yes/yes  (very small) | yes (only little) | no | 3 | *P. quasimodo*-like | in references *P. parvus* |
| SEP | 5 | 873 | 20.5 | 3.16 | 0.03 | not vaulted | ? | yes/yes/no | yes | no | 4 | *P. indicus* | in references *P. parvus* |
| PQ | 23 | 847 | 100.0 | 3.15 | 0.17 | humped | U3/U4 | yes/yes/yes | yes (many) | no | 6 | *P. quasimodo* |  |
| PI | 22 | 757 | 89.0 | 3.37 | 0.17 | not vaulted | CR | yes/yes/no | yes | yes? | 6 | *P. indicus* |  |

*Morphological identification*

Five morphospecies of the *Paracalanus parvus* complex could be identified: *Paracalanus parvus*, *Paracalanus indicus*, *Paracalanus quasimodo*, *Paracalanus tropicus* and *Paracalanus nanus* (Fig. 4). *P. nanus*, characterised by short antennules, was found in samples from the tropical Atlantic and Mediterranean. *P. tropicus* was identified from a few samples due to very high P:U ratios in the Red Sea, Indopacific and Southeast Atlantic. Specimens with characteristics of *P. quasimodo* were found in the Atlantic (including the type locality [29] near Cape Hatteras, USA), Mediterranean Sea, Northwest Pacific and Northeast Pacific. These specimens were serrated on the distal outer edge of the Exp3 of the P2-P4, the prosome was dorsally humped and the coxae of the swimming legs were decorated with spinules. However, specimens from chinese coastal waters (NWP) were more strongly ornamented than the other two groups.

*P. parvus* was identified at the type locality in the North Sea (Helgoland, Germany) and the Baltic Sea. Specimens from the Baltic Sea were slightly smaller than those from the North Sea. However, specimens with vaulted foreheads and no or only very few spinules on the posterior surfaces of the coxa of the P2-P4 were also found in the Northwest Atlantic, Southeast Atlantic, Southeast Pacific, and off southern New Zealand. For the latter, the distal outer edge of Exp3 of swimming leg 3 was serrated as in *P. indicus* (spinules very small in specimens from the Northwest Atlantic). Specimens with further characteristics (such as the spinules on the female genital segment) of *P. indicus* were found in all warm-temperate to tropical regions of the oceans.
