# Supplementary material for: Evidence of cryptic and pseudocryptic speciation in the Paracalanus parvus species complex (Crustacea, Copepoda, Calanoida)
Source: Front Zool. 2014 Mar 2;11:19. doi: 10.1186/1742-9994-11-19 (PMC3948017; doi:10.1186/1742-9994-11-19)
Supplement: Additional file 3 — Number of Cytb sequences (n), haplotypes (H) and contig sizes. MOTU: Molecular operational taxonomic unit. [file 1742-9994-11-19-S3.docx]

Supplementary Table 3: Number of Cytb sequences (n), haplotypes (H) and contig sizes. MOTU: Molecular operational taxonomic unit.

| MOTU | n | H | Contig size |
| --- | --- | --- | --- |
| PN | 5 | 4 | 245 |
| PT | 3 | 3 | 310 |
| PA | 18 | 2 | 351 |
| SEA/NZ | 22 | 6 | 351 |
| NWA | 18 | 4 | 351 |
| NEA | 6 | 5 | 300 |
| SWA | 5 | 3 | 351 |
| SWP | 3 | 3 | 351 |
| NEP | 6 | 3 | 218 |
| SEP | 3 | 2 | 351 |
| PQ | 25 | 6 | 351 |
| PI | 17 | 9 | 248 |
| Total | 131 | 50 |  |
