# Supplementary figures and images for: Evidence of cryptic and pseudocryptic speciation in the Paracalanus parvus species complex (Crustacea, Copepoda, Calanoida)
Source: Front Zool. 2014 Mar 2;11:19. doi: 10.1186/1742-9994-11-19 (PMC3948017; doi:10.1186/1742-9994-11-19)

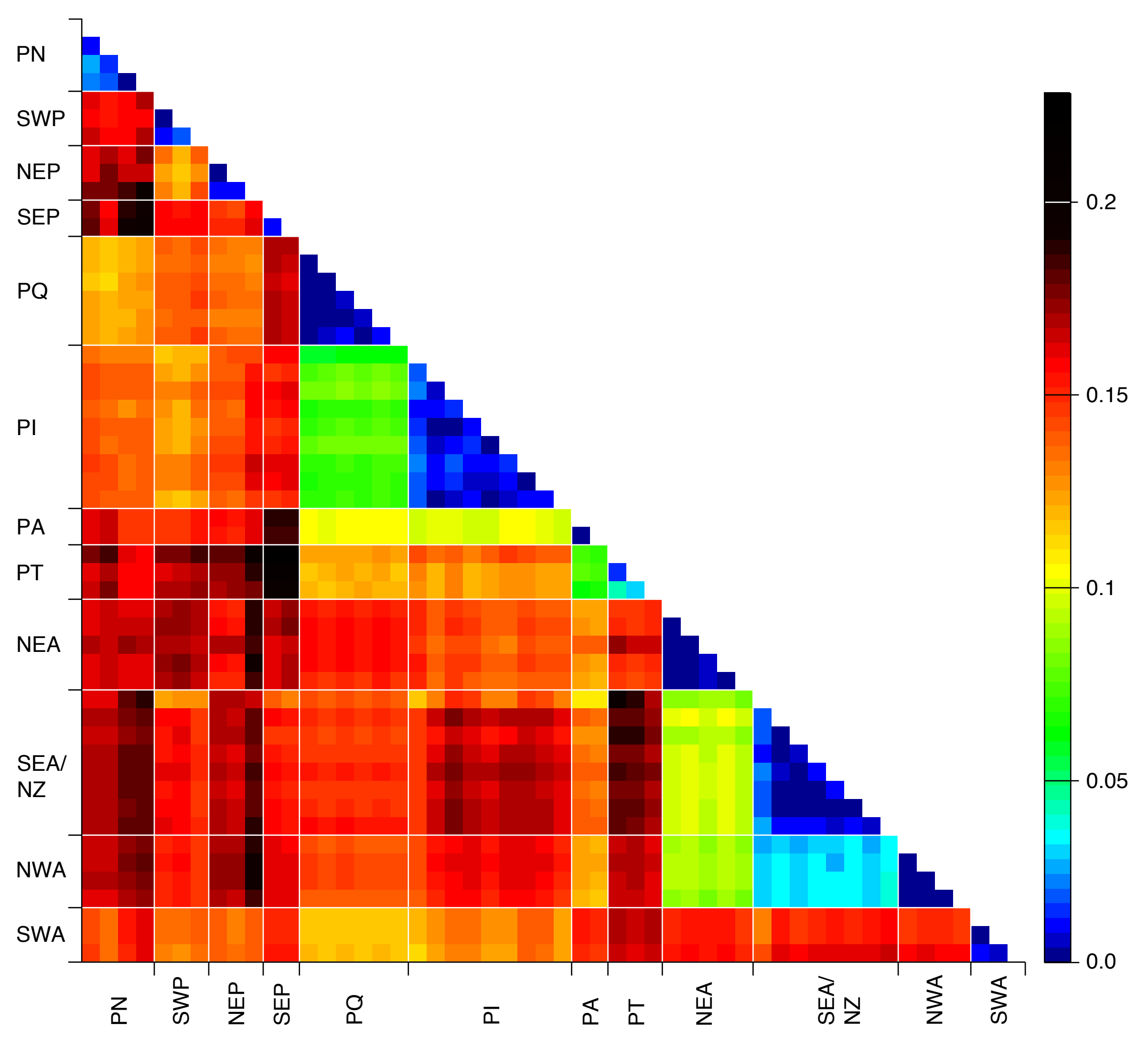

Supplement: Additional file 5 — Color heatmap representing uncorrected p-distances (Cyt b) among the haplotypes of the Paracalanus parvus species complex. [file 1742-9994-11-19-S5.pdf]
